# Cigarette Smoke Causes Caspase-Independent Apoptosis of Bronchial Epithelial Cells from Asthmatic Donors

**DOI:** 10.1371/journal.pone.0120510

**Published:** 2015-03-20

**Authors:** Fabio Bucchieri, Antonella Marino Gammazza, Alessandro Pitruzzella, Alberto Fucarino, Felicia Farina, Peter Howarth, Stephen T. Holgate, Giovanni Zummo, Donna E. Davies

**Affiliations:** 1 Academic Unit of Clinical and Experimental Sciences, Sir Henry Wellcome Laboratories, University of Southampton Faculty of Medicine, University Hospital Southampton, Southampton, United Kingdom; 2 Southampton National Institute for Health Research (NIHR) Respiratory Biomedical Research Unit, Sir Henry Wellcome Laboratories, University of Southampton Faculty of Medicine, University Hospital Southampton, Southampton, United Kingdom; 3 Dipartimento di Biomedicina Sperimentale e Neuroscienze Cliniche (BIONEC), University of Palermo, Palermo, Italy; 4 Istituto Euro-Mediterraneo di Scienza e Tecnologia (IEMEST), Palermo, Italy; 5 Institute of Biomedicine and Molecular Immunology (IBIM), Italian National Research Council (CNR), Palermo, Italy; University of Pecs Medical School, HUNGARY

## Abstract

**Background:**

Epidemiologic studies have demonstrated important links between air pollution and asthma. Amongst these pollutants, environmental cigarette smoke is a risk factor both for asthma pathogenesis and exacerbation. As the barrier to the inhaled environment, the bronchial epithelium is a key structure that is exposed to cigarette smoke.

**Objectives:**

Since primary bronchial epithelial cells (PBECs) from asthmatic donors are more susceptible to oxidant-induced apoptosis, we hypothesized that they would be susceptible to cigarette smoke-induced cell death.

**Methods:**

PBECs from normal and asthmatic donors were exposed to cigarette smoke extract (CSE); cell survival and apoptosis were assessed by fluorescence-activated cell sorting, and protective effects of antioxidants evaluated. The mechanism of cell death was evaluated using caspase inhibitors and immunofluorescent staining for apoptosis-inducing factor (AIF).

**Results:**

Exposure of PBEC cultures to CSE resulted in a dose-dependent increase in cell death. At 20% CSE, PBECs from asthmatic donors exhibited significantly more apoptosis than cells from non-asthmatic controls. Reduced glutathione (GSH), but not ascorbic acid (AA), protected against CSE-induced apoptosis. To investigate mechanisms of CSE-induced apoptosis, caspase-3 or -9 inhibitors were tested, but these failed to prevent apoptosis; in contrast, CSE promoted nuclear translocation of AIF from the mitochondria. GSH reduced the number of nuclear-AIF positive cells whereas AA was ineffective.

**Conclusion:**

Our results show that PBECs from asthmatic donors are more susceptible to CSE-induced apoptosis. This response involves AIF, which has been implicated in DNA damage and ROS-mediated cell-death. Epithelial susceptibility to CSE may contribute to the impact of environmental tobacco smoke in asthma.

## Introduction

Asthma is a chronic inflammatory disorder superimposed on remodeled airways leading to bronchial hyperresponsiveness (BHR) and variable airflow obstruction and symptoms [1]. The increased prevalence of asthma over the last 30 years is likely to be due to changes in the environment acting on a susceptible genotype both in disease induction and worsening of established disease. This proposal is supported by epidemiological studies identifying multiple interacting risk factors, including inhaled pollutants (eg. environmental tobacco smoke (ETS), particulate matter (PM_10_), oxides of nitrogen (NO_x_) and ozone (O_3_)) and respiratory virus exposure [2]. Since these agents impact on the surface of the airway, their interaction with the bronchial epithelium may translate key gene-environment effects to lead to altered inflammation, injury and repair responses in asthma [2].

The bronchial epithelium provides physical, chemical, and immunological barriers to the inhaled environment [3]. These barriers play a role in maintaining tissue homeostasis, and under appropriate conditions (eg. infection or injury) the immunological barrier becomes activated to protect the internal milieu of the lung. However, current evidence suggests that dysregulation of epithelial homeostasis can contribute to disease pathogenesis by enabling chronic activation of inflammatory and remodeling pathways. In asthma there is evidence that epithelial injury and repair is abnormal. Several studies have reported increased susceptibility to injury [4–6], and abnormal repair responses including increased expression of the epidermal growth factor receptor (EGFR) in bronchial biopsies from asthmatic adults [7] and children [8] as well as expression of the cyclin dependent kinase inhibitor, p21^waf^ [8,9]. Most recently, we have shown that the bronchial epithelial barrier is disrupted in asthma with loss of tight junctions with a consequent increase in paracellular permeability to ions and macromolecules [6]. In cultures of bronchial epithelial cells (BECs) from children, the asthmatic airway epithelium expresses more vascular endothelial growth factor at baseline [10] displays a dysregulated repair response taking longer to repair mechanically induced wounds [11] and undergoing a more extensive epithelial-mesenchymal transition in response to transforming growth factor-beta than cultures from non-asthmatic donors [12]. In adults, *in vitro* studies have identified differences between BECs from normal and asthmatic subjects in terms of epithelial repair following scrape wounding [11], their responses to respiratory virus infection [5,13] and oxidant stress [4].

The principal sources of oxidants in the bronchial airways are represented by environmental pollution and endogenously produced oxidants due to local inflammation [14]. A number of studies have indicated roles for reactive oxygen (ROS) and reactive nitrogen (RNS) species in the pathology of asthma both in terms of increased burden and decreased antioxidant defences [15] [16]. Airway responses have been shown to correlate with oxidant generation by eosinophils after antigen challenge *in vivo* [17] and neutrophil superoxide generation correlates with BHR [18]. Airway lining fluid from subjects with asthma has a lower antioxidant capacity than fluid from normal subjects [19] and the key antioxidant enzymes, superoxide dismutase (SOD) and catalase are reduced in asthma as compared to healthy individuals, with lowest levels in those patients with the most severe asthma [20]. Furthermore, reduced SOD has been found in bronchial epithelial brushings from patients with asthma and this was shown to strongly correlate with BHR [21,22].

In addition to endogenously produced ROS, environmental agents are a potent source of oxidative stress. Epidemiologic studies have demonstrated important links between air pollutants, such as diesel exhaust particles, O_3_, and ETS in asthma pathogenesis and exacerbation [23,24]; others have shown a strong link between diets low in antioxidants and asthma [25]. Exposure to cigarette smoke (CS) represents a considerable oxidant burden on the respiratory epithelium. Cigarette smoking is common in asthma and associated with poor symptom control [26]. CS facilitates allergen penetration across respiratory epithelium [27] and it activates an inflammatory cascade in the airway epithelium resulting in the production of a number of potent cytokines and chemokines, with accompanying damage to the lung epithelium, increased permeability, and recruitment of macrophages and neutrophils to the airway [28].

CS is a complex mixture of over 4,000 different compounds and high levels of oxidants and ROS have been detected in both mainstream and sidestream smoke [29]. High toxicity has been observed for at least 52 components of CS: 18 phenols, 14 aldehydes, eight *N*-heterocyclics, seven alcohols, and five hydrocarbons [30]. Most of these compounds are capable of generating ROS during their metabolism. Some lipophilic components can enter airway epithelial cells increasing intracellular ROS production by disturbing mitochondrial activity [31]. The mechanism of cigarette smoke-induced cytotoxicity is thought to incorporate oxidative stress leading to oxidative DNA damage and cell death via apoptosis and/or necrosis [32]. However failure to induce programmed cell death can result in uncontrolled cell proliferation and transformation [29].

CS is considered a major risk factor for chronic inflammatory pulmonary diseases, including asthma pathogenesis and exacerbation [28]. Furthermore, smoking is common in asthmatic individuals and it has been found to contribute to poor symptom control [26]. Since BECs from asthmatic donors are more susceptible to hydrogen peroxide-induced apoptosis [4], we hypothesized that exposure to cigarette smoke extract (CSE) would lead to increased apoptosis of BECs from asthmatic donors compared with BECs from non-asthmatic controls. We also characterized the apoptotic effects of CS, including the ability of antioxidants to protect the cells from CSE-induced apoptosis and the involvement of caspase-dependent and caspase-independent pathways.

## Materials and Methods

### Patient Characterization, Fiberoptic Bronchoscopy and Primary Bronchial Epithelial Cell Culture (PBEC)

To compare responses of PBECs from normal and asthmatic subjects, 20 subjects (10 non-asthmatic controls and 10 asthmatics) were recruited and clinically characterized (Tables 1 and 2), following ethical approval from Southampton and South West Hampshire Local Research Ethics Committee and written informed consent. Bronchial epithelial brushings were obtained by bronchoscopy using a fiberoptic bronchoscope (FB-20D; Olympus, Tokyo, Japan) in accordance with standard published guidelines (Hurd, 1991). Bronchial epithelial cells were harvested and cultured in Bronchial Epithelial Growth Medium (BEGM, Lonza, Wokingham, UK) containing 50 IU/ml penicillin and 50μg/ml streptomycin (Invitrogen, Paisley, UK), as previously described [4]. Cells were used for experimentation at passage (p)2 or 3. Control experiments confirmed that there was no significant difference between the responses of the cells at p2 or p3; the epithelial nature of cells was assessed by immunocytochemistry using a pan-cytokeratin (CK) antibody and antibodies specific for CK13 and CK18.

**Table 1:** Characteristics of all subjects used in the study.

Disease status	Asthma	Normal	P values
Number	10	10	NA
Sex (% male)	69%	60%	P = 0.6
Mean age (range)	32 (21–58)	29 (24–38)	P = 0.4
Mean (sd) FEV1% predicted^1^	77.3 (15.5)	110.3 (13.6)	P< 0.001

^1^Values for Forced Expiratory Volume in 1 second (FEV_1_) as a percentage of the predicted FEV_1_ are given as a mean and standard deviation (sd).

**Table 2:** Characteristics of subjects with asthma.

Asthma severity	Mild intermittent	Mild persistent	Moderate persistent
Number	2	5	3
Inhaled steroid use	0	4 (80%)	3 (100%)
Mean (sd) Dose ICS/day^1^	0	300 (115)	617 (256)
Mean (sd) FEV_1_% predicted^2^	92.4 (6.2)	86.9 (6.6)	77.7 (17.9)

^1^Inhaled corticosteroid (ICS) dose is given as μg of beclomethasone (BDP) used per day, expressed as the mean (sd).

^2^Values for FEV_1_ as a percentage of the predicted FEV_1_ are given as a mean and standard deviation (sd).

Prior to each treatment, PBECs were starved with Bronchial Epithelial Basal Medium (BEBM; Clonetics) containing insulin, transferrin and sodium selenite supplement (ITS; Sigma) and 1mg/ml bovine serum albumin (BSA, Invitrogen) for 24h. PBECs were treated with CSE or H_2_O_2_ (Sigma, Poole, UK) at the concentrations indicated. CSE was prepared by a modification of the method of Carp and Janoff [33]. Briefly, smoke from two Kentucky 1R4F research cigarettes (University of Kentucky, Lexington, KY) whose filters were removed was bubbled through 50 ml of BEBM for 60–70s. The resulting suspension was adjusted to pH 7.4 with concentrated NaOH, filtered through a 0.22-μM Millex-GS (Millipore, Watford, UK) filter and used immediately. When required, PBECs were pretreated with reduced glutathione (GSH, 1mM) or ascorbic acid (AA, 250nM) (both from Sigma, Poole, UK) for 30 minutes prior to the addition of CSE or H_2_O_2_. The involvement of caspases in the induction of apoptotic cell death was evaluated using the caspase-3 inhibitor, Ac-DEVD-CHO, and the caspase-9 inhibitor, Z-LEHD-FMK (both used at 120nM and obtained from BD Biosciences, Oxford, UK).

Cells were routinely photographed before and after treatment to record any morphological changes occurring in the cells.

### Analysis of Apoptosis by Fluorescence Activated cell Sorting (FACS)

Apoptosis and necrosis were measured using annexin V (AxV) and propidium iodide (PI), as previously described [4]. Briefly, after challenge PBECs were then harvested by trypsinization and combined with non-adherent cells for analysis. After washing twice in cold phosphate buffered saline (PBS), the cells were resuspended at a density of 1 x 10^5^ cells/100 μl of binding buffer (10 mM HEPES pH 7.4, 140 mM NaCl, 2.5 mM CaCl_2_) in 5 ml propylene FACS tubes. Fluorescein isothiocyanate (FITC)-conjugated AxV (1 μg/ml) and PI (2.5 μg/ml) were added to the tubes and incubated in the dark for 15 min, after which 400 μl of cold binding buffer was added and cells analyzed using a FACScan flow cytometer (Becton and Dickinson, Oxford, UK). Control tubes lacking AxV-FITC, PI, or both were included for the acquisition. Analysis of dotplots of FL1(AxV-FITC) versus FL2 (PI) was performed using WinMDI 2.8. The degree of early apoptosis was expressed as the number of AxV^+^/PI^-^ cells shown as a percentage of total cells.

### Apoptosis-inducing factor (AIF) immunofluorescent staining

After challenge with CSE ± antioxidants as described above, PBEC were fixed *in situ* in 500 μl/well of ice-cold absolute methanol and then air dried. Cells were permeabilised using 0.1% Triton X-100 (Sigma, Poole, UK) in PBS, blocked with 250μl/well of Dulbecco’s Modified Eagle’s Medium (DMEM) containing 10% foetal bovine serum (FBS) and then stained with rabbit anti-(AIF) polyclonal antibody (Ab) (clone H-300, working dilution 1:100), (Santa Cruz Biotechnology, USA). Primary Ab binding was detected using a secondary Alexa Fluor546–conjugated goat anti-rabbit Ab (1:500; Molecular Probes, USA); after washing cells were mounted with MOWIOL® containing 2.5% 1,4-diazabicyclo-octane (DABCO) anti-fade reagent and viewed using of a LEICA inverted fluorescent microscope.

### Statistical Analysis

Data were analyzed using SPSS version 11.5 for Windows (SPSS Inc, Chicago, USA). As the data were not normally distributed, the differences between the groups were analysed using non-parametric tests: differences between two dependent variables were analysed using the Wilcoxon signed rank test, differences between two independent variables using the Mann Whitney U test and for multiple comparisons, the Kruskal Wallis test. Correlations between two variables were assessed using Spearmans’ rank correlation. p< 0.05 was considered significant.

## Results

### Evaluation of the sensitivity of PBECs from normal and asthma donors to CSE

**Fig 1:**
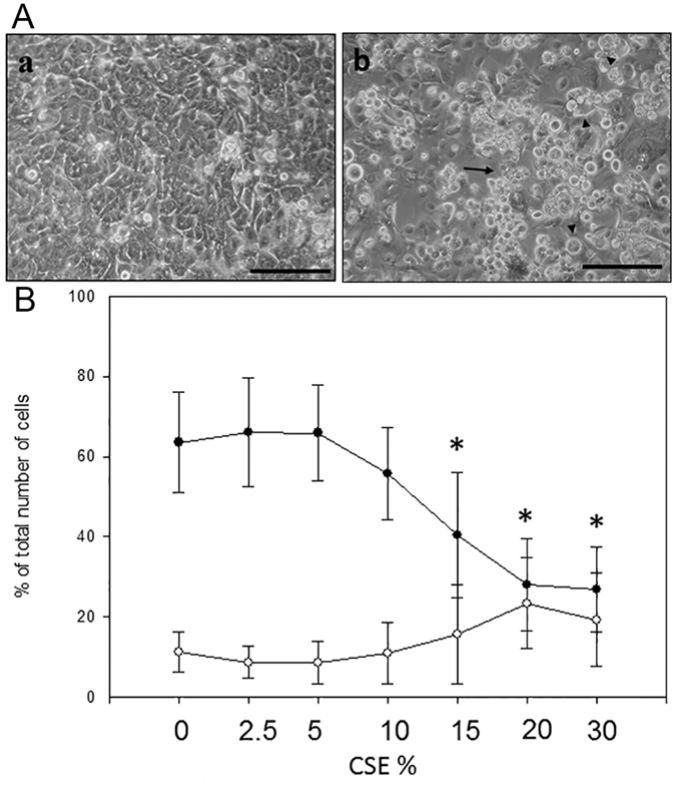
Effect of Increasing Doses of CSE on PBEC cultures. Panel A: PBEC were serum starved for 24h before being treated with serum free medium (a) or 20% CSE (b) for a further 24h. Arrows indicate condensed apoptotic cells; arrowheads swollen necrotic cells; bar = 100μm. Panel B: PBEC obtained from 4 non-asthmatic and 4 asthmatic volunteers were treated with increasing doses of CSE for 24h. Viability and EA were assessed with AxV staining. The single curve points represent median ±SD in all 8 subjects for both viability (●) and early apoptosis (○). * = (p<0.05) according to Wilcoxon Signed Rank test comparing CSE treatment with untreated control.

In a pilot experiment, PBEC were challenged with 20% CSE and morphological changes examined using phase contrast microscopy. This showed that after 24h, CSE caused many characteristic signs of necrotic and apoptotic cell death (Fig. 1a). To further characterize the cell death and the dose-dependency on these effects, PBEC obtained from 8 subjects (4 non-asthmatic and 4 asthmatic) were treated with increasing concentrations of CSE (0–30%) for 24h and adherent and non-adherent cells combined for Ax-V FACS analysis (Fig. 1b). Between 5 and 20% CSE there was a significant decline in cell viability in association with an increase in early apoptotic cells (AxV^+^/PI^-^); from these data, a dose of 20% CSE was selected for the following experiments, as it caused a significant decrease in cell viability and a significant increase in early apoptosis (EA).

**Fig 2:**
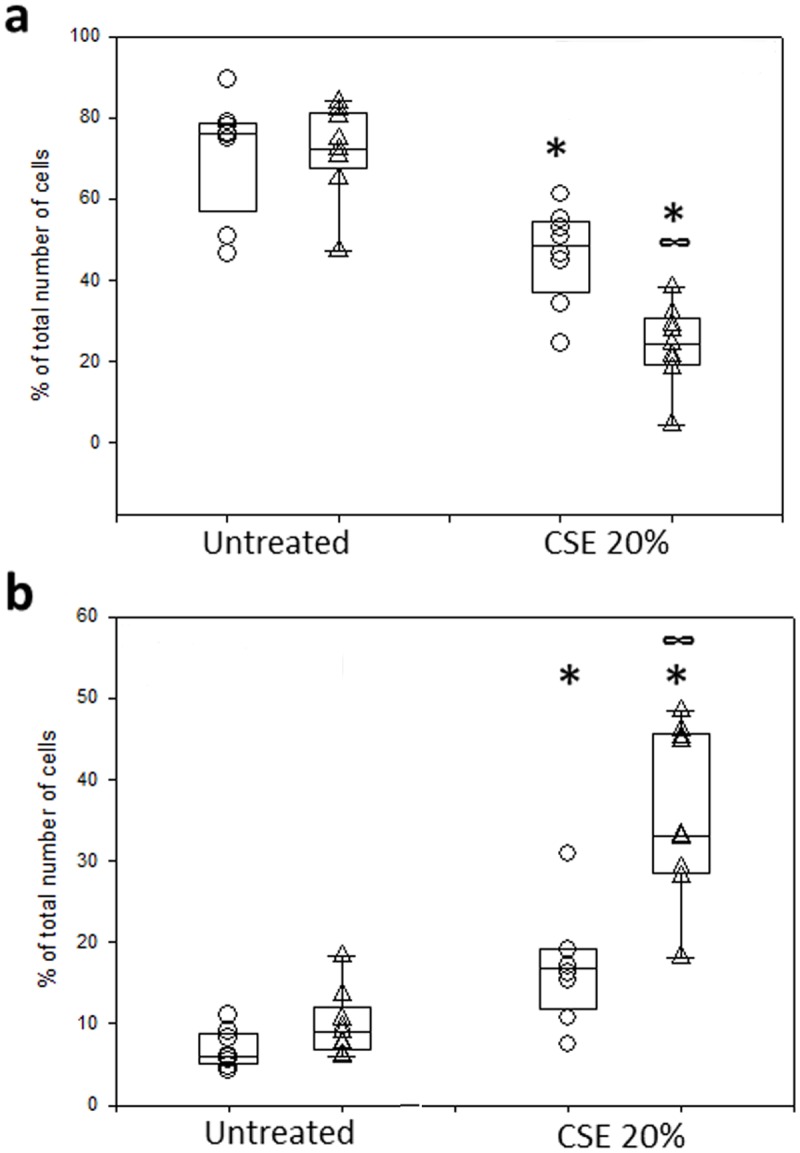
Effect of CSE on PBEC Viability and Apoptosis. PBEC obtained from non-asthmatic and asthmatic donors were treated for 24h with 20% CSE. Cell viability (a) and early apoptosis (b) were then evaluated with AnnexinV staining. The results are displayed as a box plot showing median, interquartile range and 5–95% confidence intervals using PBECs from 10 non-asthmatic (○) and 10 asthmatic (Δ) volunteers. * represents significance (p<0.05) according to Wilcoxon Signed Rank tests comparing treatment with untreated control. ∞ represents significance (p = 0.003) according to a Mann Whitney U test comparing CSE treatment between PBECs from non-asthmatic and asthmatic donors.

To compare responses of PBECs from normal and asthmatic subjects, 20 subjects (10 non-asthmatics and 10 asthmatics) were recruited (Tables 1 and 2). Cultured PBECs were challenged with 20% CSE for 24h followed by analysis of apoptosis by FACS. As shown in Fig. 2a, there was no significant difference in baseline viability between PBECs from asthmatic and non-asthmatic donors, however after treatment with 20% CSE PBECs from asthmatic donors showed an increased susceptibility to the CSE treatment compared with cells from non-asthmatic controls (% viable cells = 24.3±9.6 vs 48.5±11.9 respectively, p = 0.003). Similarly, when EA was evaluated (Fig. 2b), there was a significant increase of EA cells in the asthma PBEC group treated with CSE compared to non-asthmatic control PBECs (33.1±10.4 vs 16.7±6.9 respectively, p<0.05).

### The effect of antioxidants on CSE-induced cell death

**Fig 3:**
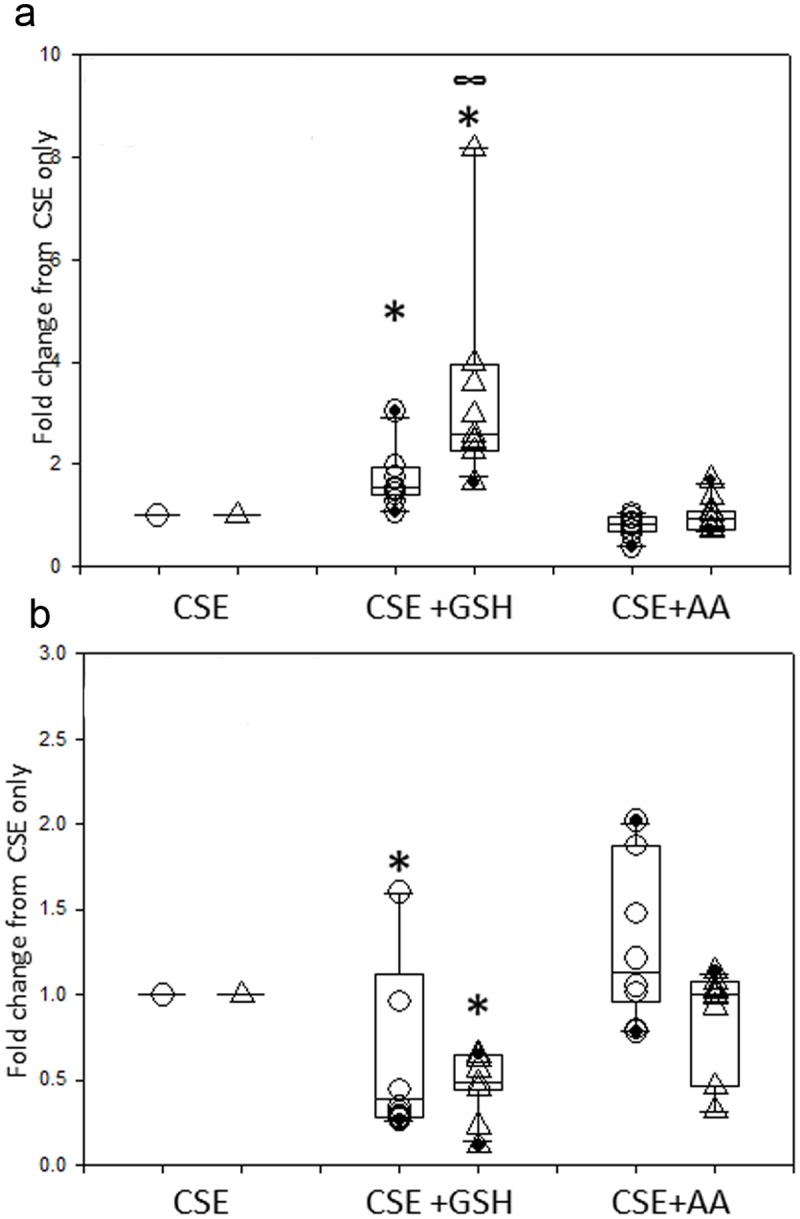
Ability of GSH, but not AA, to protect against CSE-induced cell death. PBEC obtained from non-asthmatic or asthmatic donors were treated for 24h with 20% CSE alone or in the presence of GSH (1mM) or AA (250nM). Changes in cell viability (a) and EA (b) were then evaluated with AxV staining. The results are displayed as a box plot showing median, interquartile range and 5–95% confidence intervals of fold changes from CSE alone using PBECs from 10 non-asthmatic (○) and 10 asthmatic (Δ) donors. * = p<0.05 according to Wilcoxon Signed Rank tests comparing GSH+CSE with CSE only. **∞** = p<0.05 according to a Mann Whitney U test comparing CSE+GSH treatment between PBECs from non-asthmatic and asthmatic donors.

**Fig 4:**
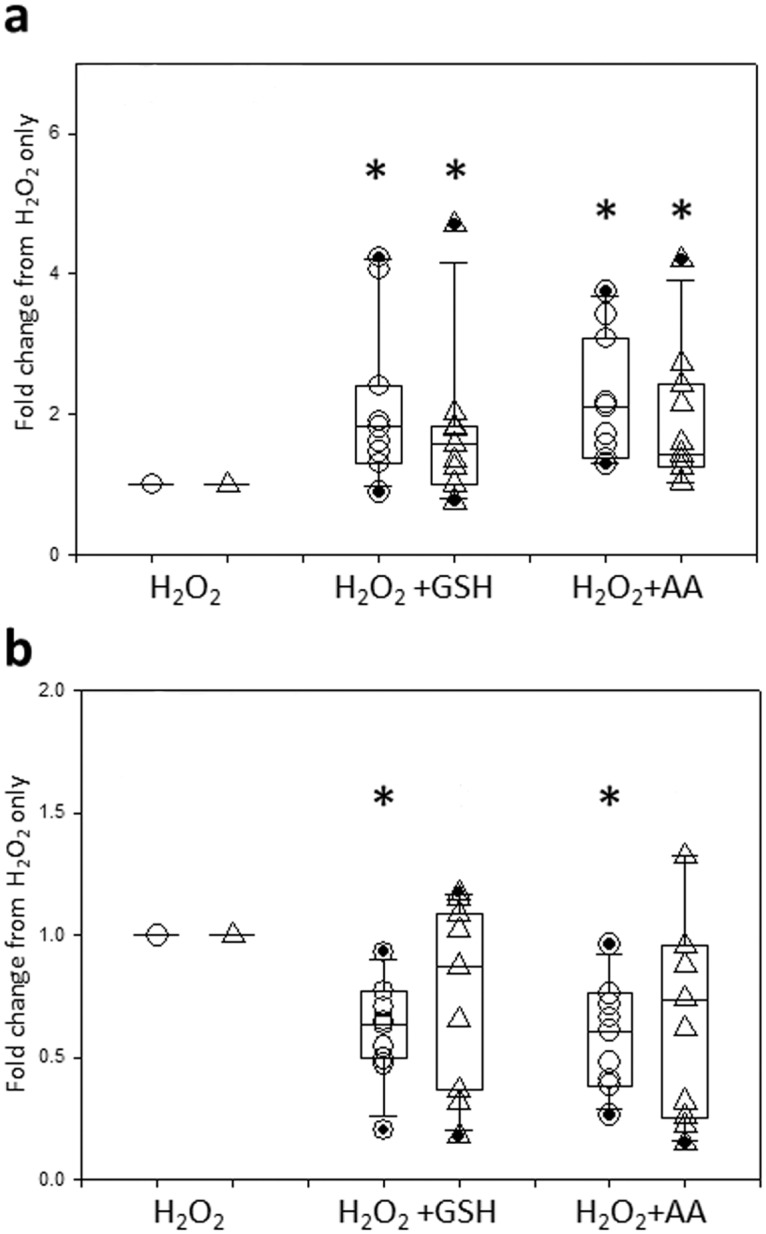
Ability of GSH and AA to protect against H_2_O_2_-induced cell death. PBEC obtained from non-asthmatic or asthmatic donors were treated for 24h with 400μM H_2_O_2_ alone or in the presence of GSH (1mM) or AA (250nM). Changes in cell viability (a) and EA (b) were then evaluated with AxV staining. The results are displayed as a box plot showing median, interquartile range and 5–95% confidence intervals of fold changes from H_2_O_2_ alone for PBECs from 10 non-asthmatic (○) and 10 asthmatic (Δ) donors. * = p<0.02 according to Wilcoxon Signed Rank tests comparing antioxidants with H_2_O_2_ only. There was no statistical difference comparing antioxidants treatment between PBECs from non-asthmatic or asthmatic donors according to a Mann Whitney U test.

To assess the protective effects of antioxidants, PBECs were treated with 250nM AA or 1mM GSH, alone or in combination with 20% CSE; in addition, 400μM H_2_O_2_ was used as a positive control for oxidant stress. Fig. 3 shows the effects of either antioxidant during CSE treatment. AA failed to protect PBECs from either non-asthmatic or asthmatic donors from CSE-induced cell death, as revealed by measures of both viability (Fig. 3a) and EA (Fig. 3b). In contrast PBECs from either healthy or asthmatic donors were significantly protected from CSE-induced cell death by GSH. Thus, GSH caused a significant increase cell viability (p = 0.005 in non-asthmatic cells and p = 0.003 in asthmatic cells) when compared to CSE alone (Fig. 3a) and, at the same time, EA levels dropped significantly (p<0.05 in non-asthmatic and p = 0.003 in asthmatic cells) (Fig. 3b). Moreover, GSH treatment caused a significantly larger (p = 0.002) overall fold-increase in viability in the asthma group when compared to the non-asthma group (Fig. 3a); this relative effect was mainly due to the larger decrease in viability caused by CSE in the PBECs from the asthmatic donors. Fig. 4 shows that in contrast with CSE treatment, both GSH and AA were able to protect the PBECs from H_2_O_2_-induced cell death. In particular, both antioxidants caused a significant (p<0.01) increase in cell viability when compared to H_2_O_2_ alone (Fig. 4a) with a concomitant decrease (p = 0.003) in early apoptosis levels in PBECs from the non-asthmatic donors (Fig. 4b); there was also a trend for a decrease in number of EA cells in PBECs from asthmatic donors, but this failed to reach statistical significance. There were no significant differences between responses of PBECs from non-asthmatic or asthmatic donors with either antioxidant.

### The role of caspases 3 and 9 in CSE-induced apoptosis

**Fig 5:**
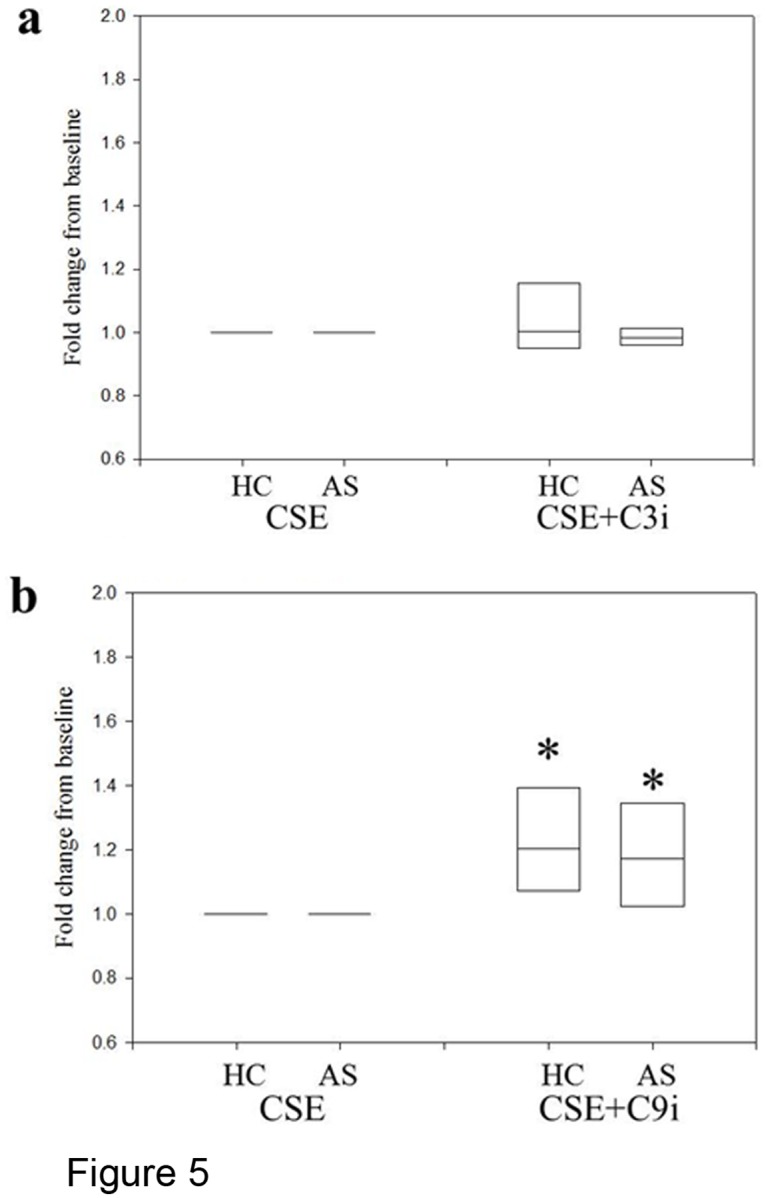
Effect of Caspase Inhibitors on CSE-Induced Apoptosis. PBEC obtained from 4 non-asthmatic and 4 asthmatic donors were treated for 24h with 20%CSE alone or in combination with Ac-DEVD-CHO (C3i) (a) or Z-LEHD-FMK (C9i) (b). Changes in EA were evaluated with AxV staining. The results are displayed as a box plot showing median and 5–95% confidence intervals of fold changes from CSE only in both groups.

To study the molecular pathways involved in CSE-induced PBEC apoptosis, PBECs obtained from 8 subjects (4 non-asthmatics and 4 asthmatics) were treated with two specific cell permeable inhibitors of caspase-3 (Ac-DEVD-CHO) and caspase-9 (Z-LEHD-FMK), two of the most relevant members of the caspase family. Before use of the inhibitor peptides, their efficacy was tested on peripheral blood lymphocytes that were stimulated to undergo apoptosis with 500nM staurosporine. Under these experimental conditions both caspase inhibitors had a significant protective effect (data not shown). PBECs were pre-treated with either caspase inhibitor 30 minutes prior the treatment with CSE, and the inhibitors were replenished after the treatment with CSE. However, as shown in Fig. 5a, b, the specific inhibition of either caspase failed to induce any significant protection from CSE-induced apoptosis in PBECs from either non-asthmatic or asthmatic donors.

### Involvement of AIF in CSE-induced Apoptosis

**Fig 6:**
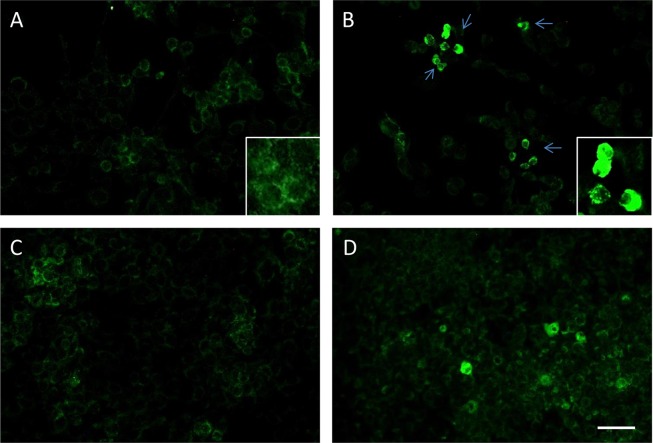
Involvement of AIF in CSE-induced Apoptosis. PBECs were left untreated (A), or were treated for 24h with 20% CSE alone (B), or with a combination of CSE and GSH (1mM) (C) or AA (250nM) (D). Cells were then washed, fixed and stained with a primary antibody directed towards AIF, followed by a secondary FITC-conjugated antibody. The figure shows that in basal conditions, PBEC expressed AIF in their cytoplasm especially inside their mitochondria (A, insert); CSE treatment caused translocation of AIF from the mitochondria to inside the nuclei (B, green arrows and insert); GSH pre-treatment caused a significant decrease in the number of nuclear-AIF positive cells whereas AA was not so effective. Bar = 30μm. Data are representative of experiments performed with PBECs from 2 donors.

As we had no evidence of caspase involvement in CSE-induced cell death, we investigated the involvement of AIF, a cell death effector molecule which operates independently of the caspase cascade and which has been implicated in DNA damage and ROS-mediated cell death [34]. Using immunofluorescent staining, AIF was detected in mitochondria under basal conditions (Fig. 6A) while CSE treatment caused its nuclear translocation (Fig. 6B). Consistent with its pro-survival effects in the presence of CSE, GSH was more effective than AA in reducing the number of nuclear-AIF positive cells (Fig. 6C and D).

## Discussion

Although many studies have demonstrated altered oxidant defenses in asthma, most appear to be a consequence of the inflammatory process. However, previous experiments carried out in our laboratory showed that PBECs from asthmatic volunteers are more susceptible to oxidant-induced apoptosis than PBECs from non-asthmatic control subjects [4]. This sensitivity of asthmatic epithelial cells to oxidant-induced apoptosis may be a key triggering mechanism that facilitates the induction and establishment of chronic inflammatory responses. Therefore, understanding more about the underlying susceptibility of epithelial cells from asthmatic subjects to environmental stress, may help to explain the why asthmatic subjects are more prone to the effects of components of the inhaled environment.

In the present study, PBECs were treated with a 20% CSE as a source of oxidative stress. After 24h of treatment with CSE, PBEC showed many characteristic signs of necrotic and apoptotic cell death. Although there was no significant difference in basal viability between PBECs from asthmatic and non-asthmatic controls, after treatment with 20% CSE, PBECs from asthmatic donors showed a significant increase in EA cells in the asthmatic group treated with CSE compared to CSE-treated non-asthmatic control cells. These findings are consistent with previous studies using fully differentiated epithelial cells from asthmatic donors, which we have shown to be more susceptible to the barrier disrupting effects of CSE [6]. In the present paper, we chose to use undifferentiated cells to evaluate the pro-apoptotic effects of CSE, as this simple model is not confounded by the additional variables of barrier integrity and altered levels of anti-oxidants in the epithelial secretions that cover fully differentiated cultures.

Microarray analysis has also shown that exposure to CSE results in up-regulation of many genes involved in oxidative stress [35]. Furthermore, applying a systems biology approach to three harmful constituents of cigarette smoke (acrolein, formaldehyde and catechol), the most prevalent toxicity mechanisms observed were DNA damage/growth arrest, oxidative stress, mitochondrial stress, and apoptosis/necrosis [36]. In order to evaluate the role of oxidative stress, we treated PBECs with AA or reduced GSH and exposed them to 20% CSE, or H_2_O_2_ as a positive control for oxidant-injury. Ax-V staining showed that the presence of AA failed to significantly protect PBECs from CSE induced apoptosis, even though it protected PBECs from normal donors against apoptosis induced by H_2_O_2_. In contrast, GSH significantly increased cell viability with a concomitant decrease in apoptosis; this protective effect against CSE was evident in PBECs from either non-asthmatic or asthmatic donors.

Free radical interactions of ROS with nucleic acids, proteins, and lipids are a major cause of cell injury because they result in a chain of free radical reactions. Ascorbate has the ability to terminate these reactions, acting as a stable donor in free radical-ROS interactions, converting into "semidehydroascorbate", a radical ion, and subsequently to dehydroascorbate both of which are relatively unreactive, and not capable of causing cellular damage [37]. GSH is a cysteine-containing tripeptide (γ-L-Glutamyl-L-cysteinylglycine); it is one of the key players in several enzymatic and non-enzymatic reactions necessary for protecting tissues against oxidative stress. The thiol portion of cysteine, in its reduced state, can donate a reducing equivalent (H^+^+ e-) to other unstable molecules (e.g. reactive oxygen compounds). Following such interaction, glutathione becomes reactive itself and creates glutathione disulfide (GSSG) by readily reacting with another reduced glutathione; this process is possible because of the relatively high concentration of glutathione in cells) [37]. The enzyme glutathione reductase regenerates GSH from GSSG. The GSH tripeptide is directly involved in the neutralization of free radicals and reactive oxygen species, as well as being able to detoxify various xenobiotics through direct conjugation. While some data suggest that the protective effect of GSH may reflect its ability to inactivate toxic substances in CSE, results obtained using A549 cells would seem to imply that volatile substances that trigger ROS generation are important contributors to the cytotoxic effects of CSE [38]. Our findings of a preferential protective effect of GSH versus AA on CSE-induced epithelial cell death are consistent with other studies showing that that CSE leads an increase in intracellular ROS and glutathione depletion however, in this previous study, cell death was apoptosis-independent, perhaps due to use of the 16HBE cell line rather than primary cells or to differences in toxicity of the CSE used [39]. Furthermore, although AA has some attenuating effects on inflammatory processes in the lungs, it is ineffective towards CS-induced symptoms [40].

Based on data using inhibitors, canonical caspase pathways were not activated in PBECs treated with either CSE or H_2_O_2._ Thus, specific inhibition of caspase-3 or-9 with Ac-DEVDCHO or Z-LEHD-FMK respectively failed to induce any significant protection from CSE-induced apoptosis in PBECs from either non-asthmatic or asthmatic donors. This is consistent with previous studies where we demonstrated that oxidative stress induced by H_2_O_2_ triggered apoptosis in a caspase- and calpain-independent manner [41]. Furthermore, we found the pro-apoptotic effects of H_2_O_2_ involved mitochondrial release of AIF and its migration into the nucleus, as observed in the present study with CSE. AIF is a flavin-binding mitochondrial intermembrane space protein that is implicated in diverse but intertwined processes that include maintenance of electron transport chain function, reactive oxygen species regulation, cell death, and neurodegeneration. AIF is involved in acute neurotoxicity provoked by trauma, hypoglycemia, transient ischemia and chronic neurodegenerative diseases [34]. It was first shown to be translocated from the mitochondria to the nucleus in response to DNA damage and excessive activation of the nuclear enzyme poly(ADP-ribose) polymerase-1 (PARP-1; EC 2.4.2.30) leading to caspase-independent cell death [42]. Similar effects have been seen in ischemia-reperfusion injury where ROS-induced DNA strand breaks lead to over-activation of PARP-1 causing depletion of NAD(+) and caspase independent cell death [43]. In non-apoptotic cells, the 67 kDa AIF precursor is cleaved by local peptidases to form a mature 57 kDa AIF molecule which is confined to the mitochondrial inter-membrane space, where it functions as an oxidoreductase. Upon induction of cell death, it migrates to the nucleus, where it contributes to chromatin condensation and the apoptotic fragmentation of DNA. In addition to this nuclear effect, cytoplasmic AIF accelerates the release of other pro-apoptotic proteins, such as cytochrome c and procaspase-9, by targeting the mitochondria. Previous studies have shown that CSE causes single-strand DNA damage in human bronchial epithelial cells and activation of PARP, but did not activate caspase 3 or cleave PARP [44]. Based on these previous observations and our current results, we postulate that in PBECs, oxidative stress-induced apoptosis does not follow the canonical caspase pathways, but rather depends on a more direct mitochondrial damage pathway; further and more detailed studies on AIF are necessary to better analyse this alternative pathway.

In conclusion, we have shown that PBECs from asthmatic donors are more susceptible to CSE-induced apoptosis and that this response involves AIF, which has been implicated in DNA damage and ROS-mediated cell-death. In view of the evidence that oxidant-antioxidant imbalance may play an important role in the pathogenesis of asthma [45] these findings may help explain the impact of environmental tobacco smoke in asthma.
